# MiR-30e-UCP2 pathway regulates alcoholic hepatitis progress by influencing ATP and hydrogen peroxide expression

**DOI:** 10.18632/oncotarget.19729

**Published:** 2017-07-31

**Authors:** Xi Jin, Mo-Sang Yu, Yue Huang, Zun Xiang, Yi-Peng Chen

**Affiliations:** ^1^ Department of Gastroenterology, The First Affiliated Hospital, School of Medicine, Zhejiang University, Hangzhou, Zhejiang, China

**Keywords:** miR-30e, UCP2, ALD, alcoholic hepatitis

## Abstract

To investigate the expression of miR-30e-UCP2 pathway in different stages of alcoholic liver disease (ALD) and its capacity and mechanism in regulating alcoholic hepatitis (AH) progress. C57BL/6 mice were fed with Lieber-DeCaril (LD) diet for 4 and 12 weeks to establish models of alcoholic fat infiltration (AFI) and AH. Based on AFI feeding, the alcoholic hepatic fibrosis (AHF) was set up with additional 4 weeks 5% carbon tetrachloride intra-abdominal injection twice per week. Serum lipid and inflammation related makers were detected while H-E staining for hepatic steatosis/ inflammation and Sirius staining for hepatic fibrosis were conducted. The apoptosis degree was tested by TUNEL plot while the hydrogen peroxide (H_2_O_2_) and ATP levels were tested by colorimetric method. MiR-30e and UCP2 over-expression were carried out by synthesizing miR-30e mimic and inserting UCP2 sequence into pCDNA3.1 plasmid. Different stages of ALD were established as indicated by increased serum TG, Tch, ALT, AST, apoptosis degree and hyaluronic acid levels as well as the typical lipid deposition, inflammatory cell infiltration and fibrosis formation in AFI, AH and AHF stages. A stepwise decreased miR-30e and increased UCP2 level was identified from AFI to AHF (p<0.05). MiR-30e over-expression significantly decreased UCP2 level. After successful miR-30e over-expression in AH, its inflammation level was decreased, followed by significantly increased ATP and H_2_O_2_ levels. Therefore, MiR-30e-UCP2 pathway participates in different stages of ALD and its therapeutic effect on AH may be through influencing oxidative stress and energy metabolism.

## INTRODUCTION

Alcoholic fatty liver disease (ALD) was regarded as one of the most critical global health problems, which comprises a spectrum of injury, including simple steatosis, acute alcoholic hepatitis, fibrosis and cirrhosis [1]. Though alcohol mediated hepatocyte injury was considered as a major cause of ALD, many other factors were also involved including insulin resistance, oxidative stress from alcohol metabolism, adipokines from visceral adipose tissue and endotoxin derived from the gut [2]. Nevertheless, the precise mechanisms of ALD are still vague. Therefore, it is meaningful to explore the pathogenesis of ALD, which would also provide novel clues for therapeutic purpose.

Currently, with the rapid development of high throughput method, the importance of post transcriptional gene regulation has been gradually recognized, especially in the area of microRNA (miRNA). MiRNA belongs to a family of noncoding RNA with the length of 19-25 nucleotides and is processed from double-stranded hairpin precursors by the RNaseIII family member Dicer. MiRNAs recognize the 3′ untranslated region of target mRNAs with imperfect complementarity, leading to translation repression in mammals and mRNA cleavage in plants [3]. MiRNAs profile have been applied in the diagnosis of various diseases, including tumors [4] and nonalcoholic fatty liver disease that we previously reported [5, 6]. Additionally, miRNA-mRNA pathways have been reported to participate in various diseases [7], including miRNA-223-IL-6-p47 [8] and miR-181b-3p-importin-a5-TLR4 [9] pathways in ALD. Nevertheless, comparing with the identified huge miRNA data, the specific miRNA-mRNA pathways in ALD are still rare and worth further investigation.

Uncoupling protein (UCP) belongs to a specific mitochondrial inner membrane protein family with the capacity of uncoupling oxidative phosphorylation [10]. We have reported the effect of various UCPs in mediating oxidative stress [11, 12], a pivotal process in the pathogenesis of ALD [13]. Therefore, UCP might be actively involved in ALD with unclear mechanisms. Furthermore, UCP2, the most widely expressed UCP in liver, was reported to be regulated by miR-133a in inflammatory bowel disease [12] and by miR-30e in kidney fibrosis [14]. Since oxidative stress has participated in the whole process of ALD, we speculated a pathogenic role of miR-30e -UCP2 pathway in ALD progression and tested this hypothesis in the ALD rat model with focusing on underlining peroxide and ATP level change.

## RESULTS

### Successful establishment of ALD in different stages

**Figure 1:**
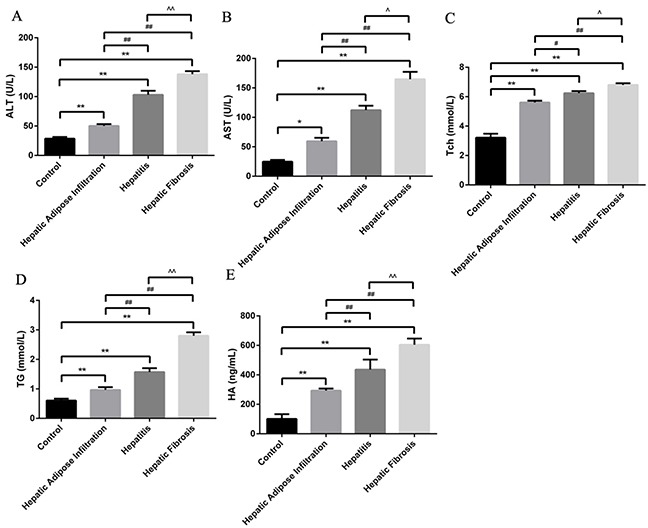
Animal model establishment of alcoholic liver disease in different stages Significantly stepwise increment of ALT **(A)**, AST **(B)**, Tch **(C)**, TG **(D)** and HA with the progression of ALD. Hepatic Adipose Infiltration represents AFL; hepatitis represents AH and hepatic fibrosis represents AHF. *, p<0.05, compared with control; *, p<0.01, compared with control; ^#^, p<0.05, compared with Hepatic Adipose Infiltration; ^##^, p<0.01, compared with Hepatic Adipose Infiltration; ^^^, p<0.05, compared with hepatitis;^^^^, p<0.01, compared with hepatitis.

**Figure 2:**
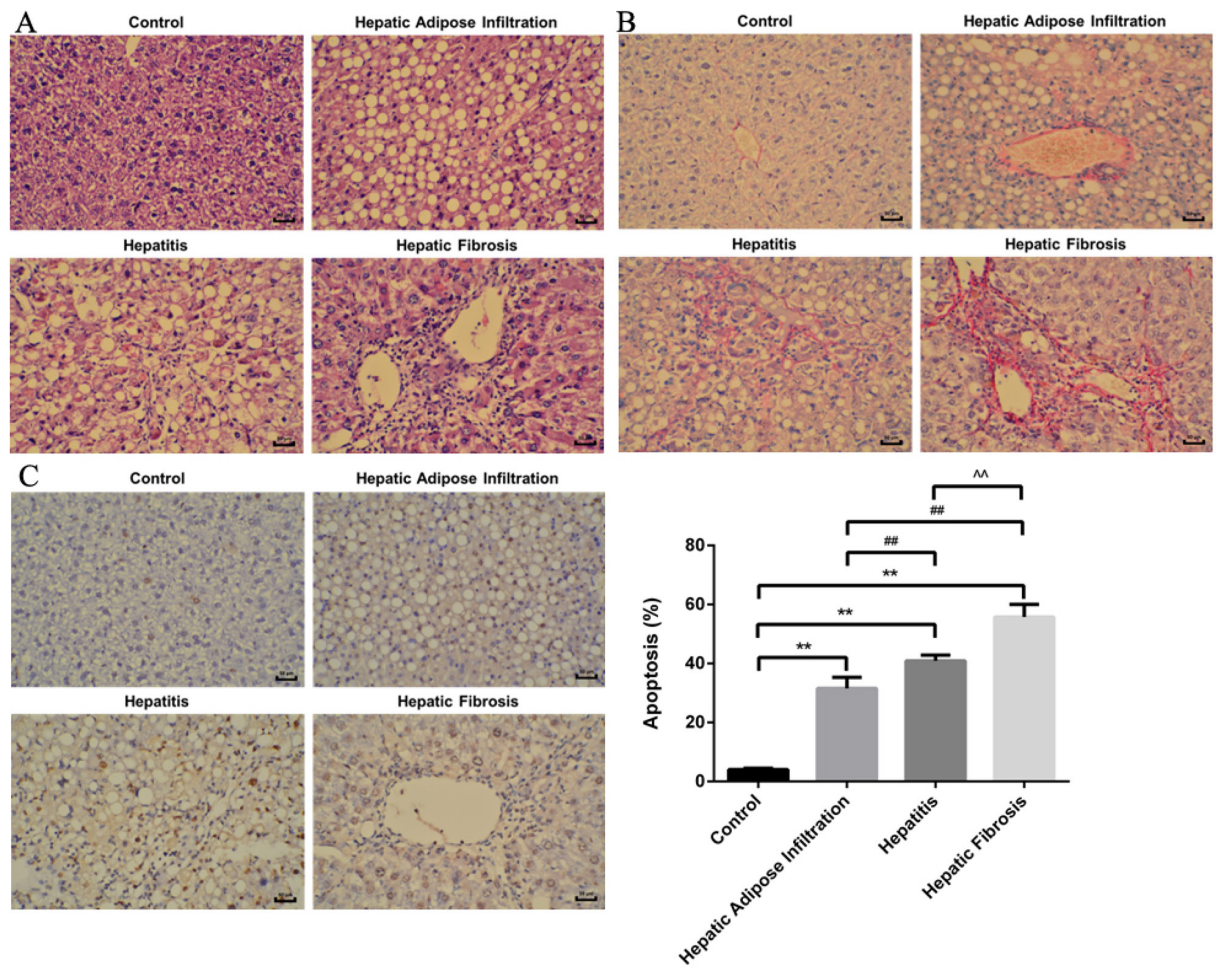
H-E, Sirius staining and TUNEL plot of ALD in different stages **(A)** H-E staining showed increased lipid deposition major in Hepatic Adipose Infiltration stage and increased inflammatory cell infiltration in hepatitis stage. **(B)** Sirius staining showed increased fibrosis formation around hepatic sinusoid as shown by red fibrosis strap deposition in hepatic fibrosis stage. **(C)** Gradually increased cell apoptosis in the whole process of ALD as shown by increased brown color cells. Hepatic Adipose Infiltration represents AFL; hepatitis represents AH and hepatic fibrosis represents AHF. *, p<0.01, compared with control; ^##^, p<0.01, compared with Hepatic Adipose Infiltration; ^^^^, p<0.01, compared with hepatitis.

Different stages of ALD rat model were successfully established, as confirmed by the serological and pathological changes. In detail, the lipid related markers (TG, Tch), the inflammation related markers (ALT, AST) and the fibrosis related marker (HA) were all significantly increased in the same tendency with ALD progression (Figure 1). Besides, the hepatic index was significantly increased in all AFI (3.52 ±0.48), AH (4.17±0.44) and AHF (4.42 ±0.51) stages, comparing with control group(2.55±0.16). Furthermore, as shown in Figure 2A, H-E staining showed increased hepatic lipid accumulation (predominantly macrovesicular) in both AFI and AH stages but decreased into normal level in AHF stage. In contrast, the chronic inflammatory cell infiltration were only observed in AH and AHF stages, with significantly increased HAI (2.52 ±0.29 in AH and 2.73 ±0.36) when comparing with the control group (0.73 ±0.07). Additional sirius staining showed the increased fibrosis formation in AHF stage (Figure 2B). Finally, TUNEL method showed the gradually significantly increased apoptosis degree in different stages of ALD, as shown by increased brown color cells in Figure 2C.

### Changes of miR-30e and UCP2 in different stages of ALD

**Figure 3:**
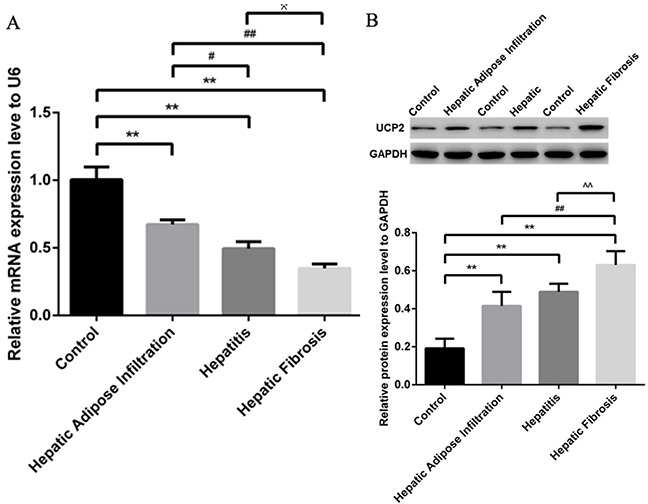
Significantly decreased miR-30e and increased UCP2 in ALD **(A)** Gradually decreased miR-30e level in different stages of ALD, as shown by qRT-PCR. **(B)** Gradually increased UCP2 level in different stages of ALD, as shown by western blot. Hepatic Adipose Infiltration represents AFL; hepatitis represents AH and hepatic fibrosis represents AHF. *, p<0.01, compared with control; ^#^, p<0.05, compared with Hepatic Adipose Infiltration; ^##^, p<0.01, compared with Hepatic Adipose Infiltration; ^^^, p<0.05, compared with hepatitis;^^^^, p<0.01, compared with hepatitis.

As shown in Figure 3A, qRT-PCR analysis showed significantly decreased miR-30e level, paralleling with the progression of ALD stages. In contrast to miR-30e change, western blot showed the significantly gradually increased UCP2 levels in different stages of ALD, starting from AFI to AH and AHF (Figure 3B). The reverse expression of miR-30e and UCP2 supported the possibility of miR-30e regulation on UCP2, as previously reported [14].

### Effect of antagonizing miR-30e and UCP2 expression in the treatment of AH

**Figure 4:**
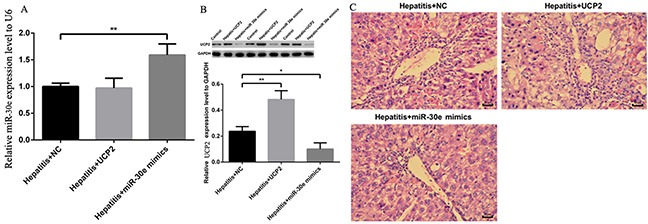
UCP2, miR-30e and H-E staining change after over expression **(A)** Significantly increased miR-30e level in rat at hepatitis stage after miR-30e over expression. **(B)** Significantly increased UCP2 level after UCP2 over expression but significantly decreased UCP2 level after miR-30e over expression. **(C)** Respectively increased and decreased inflammatory cell infiltration in UCP2 and miR -30e over expression. *, p<0.05, compared with hepatitis + NC; *, p<0.01, compared with hepatitis + NC.

AH is a vital stage of ALD, where the initiation of inflammation may trigger serial downstream reactions to promote the progression into AHF and even cirrhosis. Considering the significant change of miR-30e and UCP2 expression in different stages of ALD, we started to investigate their therapeutic effect in AH. In AH rat model, miR-30e over expression did significantly increase the miR-30e level and decrease the UCP2 level, while UCP2 over expression significantly increased UCP2 level but had no effect on miR-30e level (Figure 4A&4B). These results reinforced the possibility of upstream regulation of miR-30e on UCP2. Furthermore, respectively increased and decreased inflammatory cell infiltration after UCP2 and miR-30e over expression were observed, as presented by H-E staining (Figure 4C).

**Figure 5:**
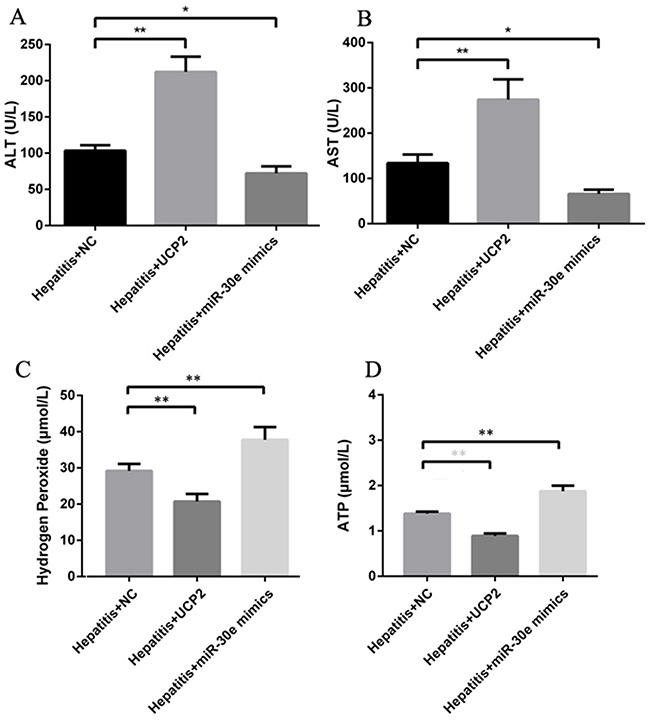
ALT, AST, H2O2 and ATP change after UCP2 and miR-30e over expression Significantly ALT **(A)** and AST **(B)** up and down regulation after respectively successful UCP2 and miR-30e over expression. Significantly hydrogen peroxide **(C)** and ATP **(D)** down and up regulation after respectively successful UCP2 and miR-30e over expression. *, p<0.05, compared with hepatitis + NC; *, p<0.01, compared with hepatitis + NC.

Except for inflammation change in pathology level, the ALT and AST levels were also increased and decreased after respective UCP2 and miR-30e over expression (Figure 5A&5B), indicating the potential therapeutic effect of miR-30e over expression in AH treatment. Furthermore, H_2_O_2_ and ATP levels were also significantly decreased and increased after respective UCP2 and miR-30e over expression, hinting the potential involvement of oxidative stress and energy metabolism in the pathogenesis of AH. Finally, UCP2 and miR-30e over expression did not significantly change TCh, TG and HA level, indicating their effect in AH may be not through regulating lipid metabolism and fibrosis formation.

## DISCUSSION

Currently, ALD has been considered as a major cause of chronic liver disease globally, with excessive alcohol intake as the major etiology but the detailed mechanism is still vague. The prevalence of ALD has been increasing in the world [15], especially in China [16] for alcohol over consumption. Therefore, it is meaningful to investigate the pathogenesis of ALD, which provide basis for novel treatment. With the development of high throughput microarray analysis, the effect of miRNA in ALD has been intensively studied [17]. Initially, the tissue miRNA profile of ALD was reported [18], followed by our findings of serum miRNA expression pattern in different stages of ALD [19]. The huge miRNA data become reservoir for in-depth miRNA-mRNA pathway research [8, 9], providing functional annotation on their effects in ALD. Another important miRNA, miRNA-122 that has the highest expression level in liver, was also found to be involved in ALD through influencing intestinal occludin expression [20].

MiR-30e was reported as a novel noninvasive biomarker for hepatocellular carcinoma [21]. Further studies demonstrated that the effect of miR-30e in liver cancer may be through targeting MTA1 for epithelial to mesenchymal transition [22] and P4HA1 for proliferation suppression [23]. However, the expression and effect of miR-30e in ALD has not been reported hitherto. Of all known UCPs, UCP2 has the highest level in liver but previous researches were mainly focusing on its association with nonalcoholic fatty liver disease, in the aspects of both expression change [24] and gene polymorphisms [25]. Nevertheless, the expression and effect of UCP2 in ALD is still unclear. Therefore, we, for the first time, successfully reported the significantly changed miR-30e and UCP2 levels in ALD. More importantly, since liver fibrosis is a pivotal stage in ALD [26], we also put it under investigation and found a stepwise change of both miR-30e and UCP2 levels with the progression from AFL to AH and AHF (Figure 3). Our results reinforced the importance of miR-30e and UCP2 in ALD.

Though the regulation of miR-30e on UCP2 was previously established [14], their association in liver is still unclear. In this study, we found that miR-30e over- expression would decrease UCP2 protein level but UCP2 over expression had no effect on miR-30e level (Figure 4A&4B), which indirectly indicates the regulation of miR-30e on UCP2. Since miR-30e-UCP2 pathway is significantly changed in ALD, we then explore its effect in AH, an important stage of ALD. After respectively antagonizing the miR-30e and UCP2 level, we found significantly changed inflammatory cell infiltration (Figure 4C) and ALT/AST level (Figure 5A&5B). Since UCP2 has the capacity of oxidative phosphorylation uncoupling, we stated to investigate its underling markers of energy metabolism and oxidative stress. We found significantly decreased and increased H_2_O_2_ and ATP levels after respective UCP2 and miR-30e over expression (Figure 5C&5D). This phenomenon was theoretically plausible since increased UCP2 may decrease the mitochondrial membrane potential, which in tern decrease ATP and H_2_O_2_ formation.

There are several limitations of this study should be acknowledged, which become further research directions. Firstly, the antagonizing of AH is only in animal model, it is better if positive *in vitro* results could be reported. Secondly, the direct regulation of miR-30e on UCP2 should be tested in cell model using Dual-luciferase reporter gene method, which may further consolidate our indirect results. Finally, it would be meaningful to detect the miR-30e and UCP2 levels in patients with different stages of ALD.

To sum up, our study, for the first time, reported the significantly dys-regulated miR-30e-UCP2 pathway in different stages of ALD. Further study also revealed the potential therapeutic effect of targeting miR-30e-UCP2 pathway in AH and the involvement of ATP and H_2_O_2_ as down stream mechanism. Our results provide novel mechanism and potential method for the pathogenesis and treatment of ALD.

## MATERIALS AND METHODS

### Ethic statement

This study was carried out in accordance with the recommendations in the Guide for the Care and Use of Laboratory Animals of the National Institutes of Health. The protocol on animal was approved by the institutional review board of the First Affiliated Hospital of Zhejiang University.

### Animal model establishment of ALD in different stages

A total of 40 Male C57BL/6J mice aged 7-8 weeks were purchased from Shilek Lab Animal (Shanghai, china). All mice received food and water ad libitum and were maintained on a 12/12-h light/dark cycle at 25°C for 7 days before any treatment. They were then randomly divided into four groups as followings: Control group (n=10) received normal diet with same amount of saline lavage and intra-abdominal injection as disease group, for 8 weeks. All disease groups were given pre-course diluted Lieber-DeCaril (LD) diet, a previously successful method for ALD establishment [27], with gradually increased alcohol concentration from 2% to 4% for 1 week as adaption feeding. The carolie composition of LD diet was alcohol 36%, protein 18%, fat 35%, carbohydrate 11% while the carolie composition of normal diet was protein 18%, fat 35% and carbohydrate 47%. Thereafter, the AFI (alcoholic fat infiltration) group (n=10) received LD diet at the 4% alcohol concentration for 4 weeks; the AH (alcoholic hepatitis) group (n=10) received the same formula of LD diet for 12 weeks; on the basis of AFI feeding, the AHF (alcoholic hepatic fibrosis) group (n=10) received additional 4 weeks 5% carbon tetrachloride intra-abdominal injection twice per week at the volume of 2ml/kg, modified from previous report [28]. All mice were sacrificed by neck dislocation with anesthesia, where blood and hepatic tissue were collected for further analysis.

### Pathology staining and laboratory test

Liver sections were routinely fixed, dehydrated and washed for further pathology staining. Hepatic index (liver wet weight/body weight) was calculated as indicated in the brackets. The Liver sections were stained with Haematoxylin-Eosin (H-E) for hepatic steaosis and inflammation observation by Olympus microscope, followed by routine Sirius staining for hepatic fibrosis according to the manufacturer's instruction (Lei-gen Biotechnology, Beijing, China). Serum triglyceride (TG), Cholesterol (Tch), alanine aminotransferase (ALT), aspartate aminotransferase (AST) and HA were tested with Hitachi 7600 clinical analyser (Department of laboratory, the First Affiliated Hospital of Zhejiang province). The severity of hepatic injury was determined by the histological activation index (HAI) as previously described [5].

### TUNEL plot, ATP and H_2_O_2_ measurement

Apoptosis in NASH animal model was accomplished by TUNEL method (100 Biotech, Hangzhou, China) as previously reported [29]. The specifically dyed apoptotic cells were observed by Olympus microscope. Usually 10 visual fields were selected and 100 cells of each field were counted, where apoptosis index=(apoptosis cell/total cell) *100%. The H_2_O_2_ level was routinely tested using a colorimetric method under the OD value of 405 nm at 37°C. The major energy product ATP was measured using a colorimetric method under the OD value of 636 nm at 37°C. All these operations were performed according to the manufacturer's instructions (Nanjing Jiancheng Bioengineering institute, China).

### Western blot and qRT-PCR

UCP2 protein level was further quantified by western blot with primary mouse polyclonal antibody raised against UCP2 (abcam, ba77363) and an ECL chemiluminescence kit (Santa Cruz, USA). The normalization was performed by blotting the same samples with a mouse anti-GAPDH antibody. For miR-30e quantitative analysis, 2μg of retrieved total RNA was reversely transcribed using stem-loop antisense primer mix and AMV transcriptase (TaKaRa, China). Real-time PCR was routinely performed on MX3000p real time PCR system (Stratagene, USA), where U6 snRNA was amplified as a normalization control. The primer sequences were as followings: For miR-30e, Sense: 5′-ACACTCCAGCTGGGTGTAAACATC CTTGAC-3′, Antisense: 5′-CTCAACTGGTGTCGTGGAGTCGGCAA TTCAGTTG AGCTTCCA-3′; For U6, Sense: 5′-CTCGCT TCGGCAGCACA-3′, Antisense: 5′-A ACGCTTCA CGAATTTGCGT-3′. Finally, the relative amount of miR-30e to U6 and UCP2 to GAODH were calculated using the equation 2^−^CT^, where ^CT = C_TmiRNA/UCP2_-C_Tu6/GAPDH_.

### UCP2 and miR-30e over expression in ASH model

UCP2 sequence was firstly amplified with the following primer: F-CCCAAGCTT ATGGTTGG TTTCAAGGCCACAG; R-CCGGAATTCTCAGAAAG GTGCCTCCC GAGAT, followed by double enzyme digestion in the sites of HindIII and ECORI and inserted into the Plasmid pCDNA3.1(+). Thereafter, the pCDNA3.1-UCP2 plasmid was sequentially transfected into competent cell, verified by qRT-PCR and finally harvested for further *in-vivo* injection. Besides, miR-30e was synthesized and provided by a commercial company (Ji-ma Biotechnology, Shanghai, china) with the following sequence: Sense, 5′ UGUAAACAUCCUUGACUGGAAG 3′; Anti-sense, 5′ UCCAG UCAAGGAUGUUUACAUU 3′. The pCDNA3.1(+)-UCP2 plasmid and miR-30e mimic were utilized to transfect ASH animal model by caudal vein injection once a week as an supplementary treatment with ALD establishment. Therefore, the groups were categorized as followings: ASH group (n=5), ASH+UCP2 group (n=5) and ASH +miR-30e mimic group (n=5).

### Statistics

Statistical analyses were performed using SPSS, version 16 (Chicago, IL, USA). Data are presented as the mean ± standard deviation (SD) when found to be normally distributed or as the median if the distribution was skewed. Differences between groups were analyzed using the Student's *t*-test or the Mann–Whitney *U* test. P<0.05 was considered with statistical significance.

## References

[1] Chacko KR, Reinus J (2016). Spectrum of alcoholic liver disease. Clin Liver Dis.

[2] Sugimoto K, Takei Y (2017). Pathogenesis of alcoholic liver disease. Hepatol Res.

[3] Ambros V (2004). The functions of animal microRNAs. Nature.

[4] Calin GA, Croce CM (2006). MicroRNA signatures in human cancers. Nat Rev Cancer.

[5] Jin X, Ye YF, Chen SH, Yu CH, Liu J, Li YM (2009). MicroRNA expression pattern in different stages of nonalcoholic fatty liver disease. Dig Liver Dis.

[6] Jin X, Chen YP, Kong M, Zheng L, Yang YD, Li YM (2012). Transition from hepatic steatosis to steatohepatitis: unique microRNA patterns and potential downstream functions and pathways. J Gastroenterol Hepatol.

[7] Moreno-Moya JM, Vilella F, Simon C (2014). MicroRNA: key gene expression regulators. Fertil Steril.

[8] Li M, He Y, Zhou Z, Ramirez T, Gao Y, Gao Y, Ross RA, Cao H, Cai Y, Xu M, Feng D, Zhang P, Liangpunsakul S, Gao B (2017). MicroRNA-223 ameliorates alcoholic liver injury by inhibiting the IL-6-p47phox-oxidative stress pathway in neutrophils. Gut.

[9] Saikia P, Bellos D, McMullen MR, Pollard KA, de la Motte C, Nagy LE (2017). miR181b-3p and its target importin alpha5 regulate TLR4 signaling in Kupffer cells and liver injury in mice in response to ethanol. Hepatology.

[10] Jin X, Xiang Z, Chen YP, Ma KF, Ye YF, Li YM (2013). Uncoupling protein and nonalcoholic fatty liver disease. Chin Med J.

[11] Jin X, Yang YD, Chen K, Lv ZY, Zheng L, Liu YP, Chen SH, Yu CH, Jiang XY, Zhang CY, Li YM (2009). HDMCP uncouples yeast mitochondrial respiration and alleviates steatosis in L02 and hepG2 cells by decreasing ATP and H2O2 levels: a novel mechanism for NAFLD. J Hepatol.

[12] Jin X, Chen D, Zheng RH, Zhang H, Chen YP, Xiang Z (2017). miRNA-133a-UCP2 pathway regulates inflammatory bowel disease progress by influencing inflammation, oxidative stress and energy metabolism. World J Gastroenterol.

[13] Galicia-Moreno M, Gutierrez-Reyes G (2014). The role of oxidative stress in the development of alcoholic liver disease. Rev Gastroenterol Mex.

[14] Jiang L, Qiu W, Zhou Y, Wen P, Fang L, Cao H, Zen K, He W, Zhang C, Dai C, Yang J (2013). A microRNA-30e/mitochondrial uncoupling protein 2 axis mediates TGF-beta1-induced tubular epithelial cell extracellular matrix production and kidney fibrosis. Kidney Int.

[15] Masarone M, Rosato V, Dallio M, Abenavoli L, Federico A, Loguercio C, Persico M (2016). Epidemiology and natural history of alcoholic liver disease. Rev Recent Clin Trials.

[16] Li YM, Fan JG, Wang BY, Lu LG, Shi JP, Niu JQ, Shen W, Chinese Association for the Study of Liver Disease (2011). Guidelines for the diagnosis and management of alcoholic liver disease: update 2010: (published in Chinese on Chinese Journal of Hepatology 2010; 18: 167-170). J Dig Dis.

[17] Szabo G, Satishchandran A (2015). MicroRNAs in alcoholic liver disease. Semin Liver Dis.

[18] Dolganiuc A, Petrasek J, Kodys K, Catalano D, Mandrekar P, Velayudham A, Szabo G (2009). MicroRNA expression profile in Lieber-DeCarli diet-induced alcoholic and methionine choline deficient diet-induced nonalcoholic steatohepatitis models in mice. Alcohol Clin Exp Res.

[19] Chen YP, Jin X, Xiang Z, Chen SH, Li YM (2013). Circulating MicroRNAs as potential biomarkers for alcoholic steatohepatitis. Liver Int.

[20] Zhao H, Zhao C, Dong Y, Zhang M, Wang Y, Li F, Li X, McClain C, Yang S, Feng W (2015). Inhibition of miR122a by Lactobacillus rhamnosus GG culture supernatant increases intestinal occludin expression and protects mice from alcoholic liver disease. Toxicol Lett.

[21] Bhattacharya S, Steele R, Shrivastava S, Chakraborty S, Di Bisceglie AM, Ray RB (2016). Serum miR-30e and miR-223 as novel noninvasive biomarkers for hepatocellular carcinoma. Am J Pathol.

[22] Deng L, Tang J, Yang H, Cheng C, Lu S, Jiang R, Sun B (2017). MTA1 modulated by miR-30e contributes to epithelial-to-mesenchymal transition in hepatocellular carcinoma through an ErbB2-dependent pathway. Oncogene.

[23] Feng G, Shi H, Li J, Yang Z, Fang R, Ye L, Zhang W, Zhang X (2016). MiR-30e suppresses proliferation of hepatoma cells via targeting prolyl 4-hydroxylase subunit alpha-1 (P4HA1) mRNA. Biochem Biophys Res Commun.

[24] Li L, Chen J, Ni Y, Feng X, Zhao Z, Wang P, Sun J, Yu H, Yan Z, Liu D, Nilius B, Zhu Z (2012). TRPV1 activation prevents nonalcoholic fatty liver through UCP2 upregulation in mice. Pflugers Arch.

[25] Mohseni F, Farajnia S, Farhangi MA, Khoshbaten M, Jafarabadi MA (2017). Association of UCP2 −866G>A polymorphism with nonalcoholic fatty liver disease in patients from North-West of Iran. Lab Med.

[26] Bataller R, Gao B (2015). Liver fibrosis in alcoholic liver disease. Semin Liver Dis.

[27] Cao YW, Jiang Y, Zhang DY, Wang M, Chen WS, Su H, Wang YT, Wan JB (2015). Protective effects of Penthorum chinense Pursh against chronic ethanol-induced liver injury in mice. J Ethnopharmacol.

[28] Geng Y, Sun Q, Li W, Lu ZM, Xu HY, Shi JS, Xu ZH (2016). The common dietary flavonoid myricetin attenuates liver fibrosis in carbon tetrachloride-treated mice. Mol Nutr Food Res.

[29] Jin X, Liu J, Chen YP, Xiang Z, Ding JX, Li YM (2017). Effect of miR-146 targeted HDMCP up-regulation in the pathogenesis of nonalcoholic steatohepatitis. PLoS One.

